# Deep-Motion-Net: GNN-based volumetric liver shape reconstruction from single-view 2D projections

**DOI:** 10.1007/s11548-026-03685-1

**Published:** 2026-05-13

**Authors:** Isuru Wijesinghe, Michael Nix, Arezoo Zakeri, Alireza Hokmabadi, Bashar Al-Qaisieh, Ali Gooya, Zeike Taylor

**Affiliations:** 1 Centre for Computational Imaging and Simulation Technologies in Biomedicine, School of Mechanical Engineering, University of Leeds, Leeds, UK; 2 Department of Medical Physics and Engineering, St James’s University Hospital, Leeds Teaching Hospitals NHS Trust, Leeds, UK; 3 Division of Informatics, Imaging, and Data Sciences, Faculty of Biology, Medicine and Health, University of Manchester, Manchester, UK; 4 School of Medicine and Population Health, University of Sheffield, Sheffield, UK; 5 School of Computing Science, University of Glasgow, Glasgow, UK

**Keywords:** Motion modelling, Adaptive radiotherapy, Graph neural network, Graph attention, X-ray image, Synthetic data

## Abstract

**Purpose:**

Internal anatomical motion challenges precise radiation delivery during external beam radiotherapy. Estimating and compensating for anatomical motion are essential for improving planned dose delivery to target volumes while sparing organs-at-risk. This research achieves accurate motion prediction using only planar X-ray imaging from conventional linear accelerators, without surrogate signals or invasive fiducial markers.

**Methods:**

We propose Deep-Motion-Net: a patient-specific end-to-end graph neural network (GNN) enabling 3D volumetric organ reconstruction from single in-treatment kV planar X-ray images at arbitrary projection angles. A 2D convolutional neural network (CNN) encoder extracts image features, which four feature pooling networks fuse to a 3D template organ mesh. A ResNet-based graph attention network then deforms the feature-encoded mesh. Training uses synthetically generated organ motion instances and corresponding kV images, created by deforming a reference CT volume aligned with the template mesh, generating digitally reconstructed radiographs (DRRs) at required angles, and applying DRR-to-kV style transfer via conditional CycleGAN.

**Results:**

Quantitative testing on synthetic respiratory motion scenarios and qualitative assessment on in-treatment images from four liver cancer patients demonstrated overall mean prediction errors of 0.16 ± 0.13 mm, 0.18 ± 0.19 mm, 0.22 ± 0.34 mm, and 0.12 ± 0.11 mm across datasets. Mean peak prediction errors were 1.39 mm, 1.99 mm, 3.29 mm, and 1.16 mm.

**Conclusion:**

This approach leverages accessible in-treatment imaging, avoiding expensive MRI systems or invasive markers. To the best of our knowledge, this is the first deep learning framework reconstructing volumetric 3D organ models from single-view images at arbitrary angles throughout an entire in-treatment scan series. Our approach achieves sub-millimetre accuracy when validated on synthetic motion instances and demonstrates clinical feasibility on real-treatment kV images, for which volumetric ground truth is inherently unavailable. The code is available at https://github.com/isurusuranga/DeepMotionNet.

**Supplementary Information:**

The online version contains supplementary material available at 10.1007/s11548-026-03685-1.

## Introduction

Internal anatomical motion during external beam radiotherapy confounds precise radiation delivery to target volumes, which is critical for achieving tumour coverage while preserving surrounding tissues [1]. Unaccounted motion can cause overdosing of organs-at-risk (OARs) or underdosing of tumours, leading to poorer survival and increased morbidity [1]. This challenge is particularly acute in hypo-fractionated treatments where higher, precisely targeted doses are essential.

While cone beam CT acquired at treatment start ensures correct patient positioning, this static representation ignores subsequent anatomical motion during radiation delivery. To address respiratory-induced motion, various mitigation strategies exist [1, 2]. Passive approaches like internal target volumes (ITVs) with motion-encompassing margins [2] necessitate greater normal tissue irradiation [3] and reduced radiation intensity. Active methods—including respiratory gating and real-time tracking—require real-time tumour position information but offer better outcomes. Gating prolongs treatment sessions by delivering radiation only during specific respiratory phases [1], while tracking repositions the beam continuously but is technically challenging. Active motion mitigation methods often rely on invasive implanted markers providing only point-based information rather than complete target/OAR geometry [4]. Noninvasive imaging is preferred. MR-linacs provide excellent real-time, radiation-free images with superior soft tissue contrast [5], but only deliver orthogonal 2D slices, are expensive, and remain inaccessible to most patients.

Conventional linacs equipped with on-board kV X-ray imaging present an alternative, though challenges include poor soft tissue contrast and volumetric information compression. Deep learning advances show promise: Ying et al. [6] reconstructed 3D CT from biplanar X-rays; Wang et al. [7] predicted lung surfaces from single-view projections using deformation; Tong et al. [8] combined CNN–GNN to reconstruct liver meshes with mean shape priors, later extended to multiple abdominal organs [9, 10]. However, these methods operate only at fixed projection angles. Wei et al. [11] addressed this using CNN-predicted PCA coefficients in 4D CT motion models with angle-dependent layers, but faced challenges with DRR–CBCT intensity variations and discrete angle handling. Shao et al. [12] proposed GNN-based liver surface prediction with finite element volumetric deformation, introducing biomechanical constraints but requiring per-angle retraining and 3840 feature vector per node extracted from a ResNet-50 backbone. Critically, existing methods [7–10, 12] share key limitations: (1) fixed rather than arbitrary projection angles [7–10, 12]; (2) surface-only reconstruction without volumetric deformations [7–11]; and (3) wide field-of-view (FOV) requirements incompatible with clinical practice [7–10, 12].

We present Deep-Motion-Net, a deep GNN estimating 3D volumetric organ deformation from single kV planar X-ray images at arbitrary gantry angles. The model learns mappings from CNN-extracted features to mesh node displacements via graph attention (GAT) networks. Feature pooling networks enable end-to-end training by fusing image features with 3D graph nodes, while projection angle-dependent features are learned through angle encoding as additional input channels. To our knowledge, this is the first framework reconstructing 3D anatomy from arbitrary-angle, limited-FOV kV projections.

## Methods

Our approach uses a GNN that learns mappings from kV image features to nodal displacements of patient-specific organ meshes, with individual training per patient.

### 3D organ shape representation

Volumetric organ shape is represented using 3D unstructured tetrahedral meshes, described as an undirected graph $G = {V, E, F}$ with vertices *V*, edges *E*, and vertex features *F*. Patient-specific template meshes were constructed from reference CT volumes, with vertices N=785, 827, 803, and 756, respectively.

### Model architecture

**Fig. 1:**
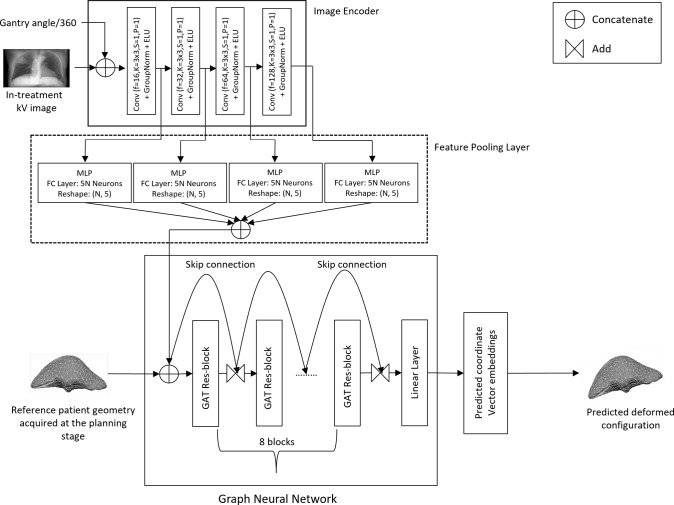
The Deep-Motion-Net architecture uses a 2D CNN image encoder to extract projection angle-dependent semantic features from input kV X-ray images. Four learnable feature pooling networks attach these features to vertices in the patient-specific template mesh, and a graph-attention-based network predicts the corresponding mesh deformation

The proposed architecture (shown in Fig. 1) consists of two components: a 2D CNN encoder with four learnable feature pooling networks (FPNs), which extract perceptual features from the kV image and attach them to mesh nodes and a GAT-based mesh deformation network.

**Incorporating projection angle**: The 2D CNN encoder receives a single-view kV image and its projection angle. Since the anatomical information visible in 2D projections varies across gantry angles, explicitly encoding the projection geometry allows the model to resolve these perspective-dependent features and accurately map them to the 3D volumetric space during continuous gantry rotation. To facilitate this, the projection angle is normalised by dividing by 360 (gantry rotation range: 0–360 degrees) and concatenated as an extra channel to the input image after expanding and reshaping to match the image spatial resolution (256 × 256). While the projection angle is a global parameter constant across the entire projection, we encode it as a full 256 × 256 channel where each pixel contains the same normalised angle value. This encoding strategy ensures the angle information is spatially available to all convolutional operations throughout the hierarchical feature extraction process, allowing the network to learn angle-dependent feature representations at multiple scales.

**2D CNN configuration:** The image encoder extracts projection angle-dependent perceptual features using four convolutional layers with 16, 32, 64, and 128 filters, respectively. All convolutional layers use 3 × 3 kernels with stride and padding of 1. Exponential linear units (ELUs) provide nonlinearity after each convolution. Hidden-layer outputs are normalised using group normalisation [13] to reduce internal covariate shift during training. Output feature maps from each convolutional layer are down-sampled via 2 × 2 max-pooling with stride 2 before passing to the next layer.

**Feature pooling networks:** Learnable FPNs were integrated to associate image features with mesh nodes, enabling end-to-end training and improving graph network performance. Four FPNs, each linked to a CNN layer (Fig. 1), consisted of adaptive average pooling (7×7 output) followed by a fully connected (FC) layer with 5*N* neurons. After flattening, the FC layer with 5*N* neurons produces outputs reshaped to (*N*, 5) to facilitate the direct attachment of latent features to individual mesh vertices. The resulting outputs (*N*, 5) were concatenated with 3D mesh coordinates, giving each vertex 23 features (20 from FPNs and 3 representing 3D position).

**Mesh deformation network**: This network estimates 3D coordinates of deformed mesh vertices using a GNN composed of GAT-based residual blocks. Residual connections enhance training efficiency and output quality by preserving information from earlier layers. Each block follows the residual bottleneck design [14], replacing 1 × 1 and 3 × 3 convolutions with per-vertex FC and GAT layers [15]. Group normalisation [16] is used instead of batch normalisation to mitigate errors from small batch sizes, and ELU activations provide nonlinearity. This design extends the graph CNN of [17], with two modifications: graph convolutional network (GCN) layers are replaced by GAT layers, which assign attention-based, non-uniform weights to neighbouring nodes (unlike isotropic GCN filtering [18]), and ReLU is replaced with ELU to avoid dying neurons and allow negative outputs. Ablation studies were conducted as described in Section 5 of Online Resource 1.

### Loss functions

The overall objective function $\mathcal {L}$ is defined as:

1 $$\begin{aligned} \mathcal {L} = \mathcal {L}_{Shape} + \lambda \mathcal {L}_{Laplacian}, \end{aligned}$$

with weighting term λ. $\mathcal {L}_{Shape}$ quantifies the difference between the predicted and ground-truth 3D meshes. The template mesh starting shape is defined by its vertices *V* (Sect. 3D organ shape representation). We define *Y* and $\hat{Y}$ to be the ground-truth and predicted deformed positions of these vertices. An intuitive objective is then to minimise the per-vertex $L_{1}$ loss between *Y* and $\hat{Y}$:

2 $$\begin{aligned} \mathcal {L}_{Shape} = \sum _{i=1}^{N} \left\Vert {\hat{Y}_{i} - Y_{i}}\right\Vert _{1}, \end{aligned}$$

where $Y_{i}$ and $\hat{Y}_{i}$ are the $i^\mathrm{{th}}$ vertices in the respective sets.

**Table 1:** Summary statistics for all test sets with synthetic kV images. Patient case numbers are in column 1. $E_{pred}$ and $U_{GT}$ denote prediction errors and ground-truth nodal deformation magnitudes, respectively. *Mean (std)*: means (standard deviations) across all nodes, deformation states, and projection angles. *Mean peak*: means of peak values per deformation state across all projection angles. *Max peak*: overall maximum values across all nodes, states, and angles. 99$^{th}$ Percentile: $99^\textrm{th}$ percentile values across all nodes, states, and angles. All values in mm. **Bold** values indicate prediction errors that fall within the clinically acceptable liver SBRT margin of 5.0 mm. Although isolated maximum peak errors exceed this margin in some cases, the 99$^\textrm{th}$ percentile values remain well below 2 mm, indicating that large errors are highly localised and not representative of typical model performance

Case		Mean (std)	Mean peak	Max peak	$99^\textrm{th}$ Percentile
1	$E_{\text {Pred}}$	0.16±0.13	1.39	6.75	0.75
	$U_{\text {GT}}$	10.18±1.33	14.71	28.12	13.85
2	$E_{\text {Pred}}$	0.18±0.19	1.99	7.97	0.97
	$U_{\text {GT}}$	11.65±1.76	15.76	34.91	14.38
3	$E_{\text {Pred}}$	0.22±0.34	3.29	14.66	1.81
	$U_{\text {GT}}$	14.89±2.54	19.36	49.63	17.65
4	$E_{\text {Pred}}$	0.12±0.11	1.16	4.36	0.51
	$U_{\text {GT}}$	10.07±1.13	12.86	25.64	11.97

**Fig. 2:**
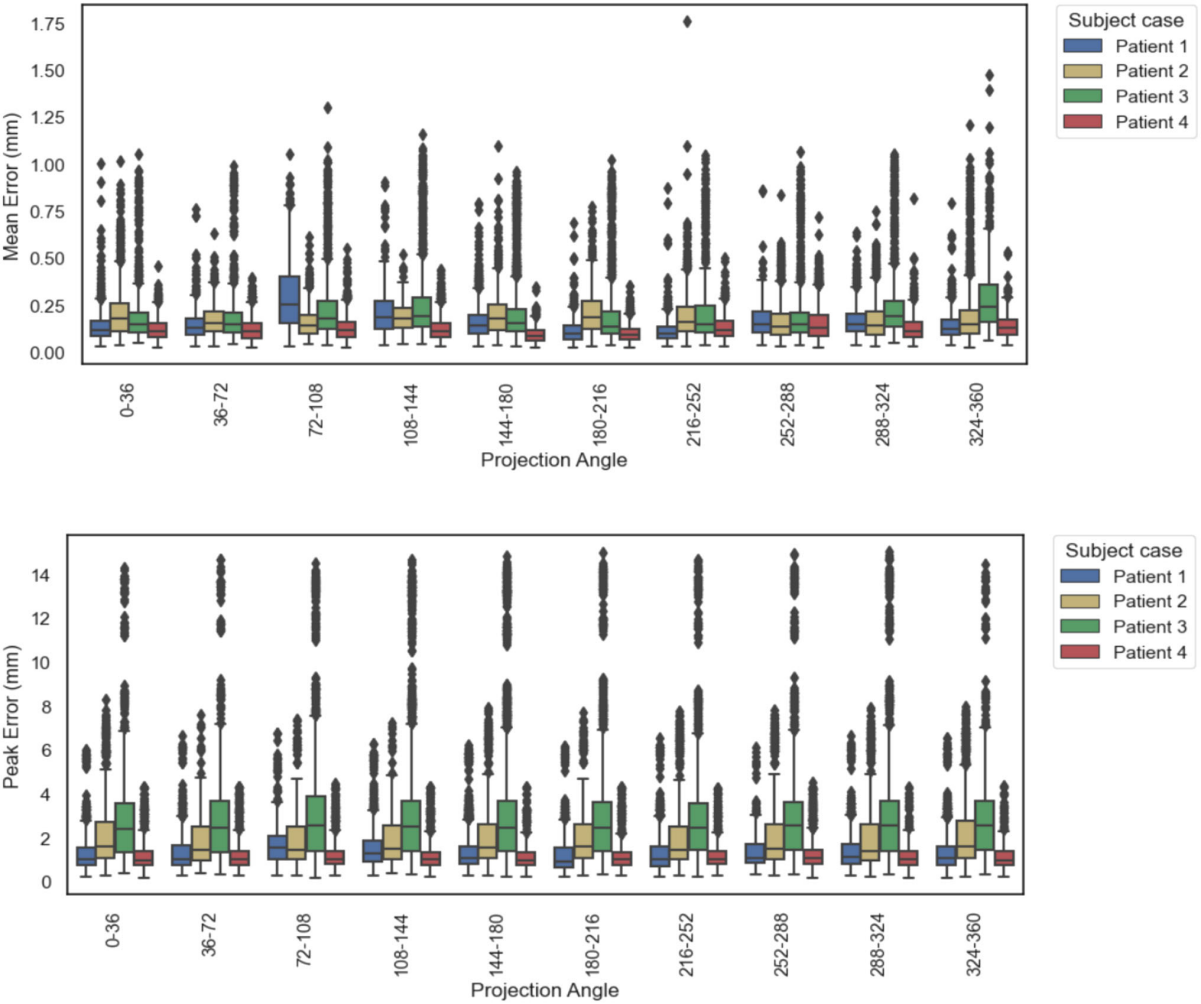
Effect of projection angle on prediction accuracy: box-and-whisker plots of mean (top) and peak (bottom) prediction errors grouped according to image projection angle (degrees). Each box and whisker shows the distribution of errors for the indicated projection angle using all deformation states in the test set. For clarity of visualisation, angles are further grouped into 10 equal bins covering a full revolution. Results for patients 1 (blue), 2 (yellow), 3 (green), and 4 (red) are shown for each bin

Using $\mathcal {L}_{Shape}$ alone the vertices were found to move too freely. We introduced a discrete Laplacian ([19]) loss $\mathcal {L}_{Laplacian}$ as a regularisation term to limit this freedom by ensuring vertices do not move too far in relation to their neighbours. The additional constraint ensures that the mesh has a smooth surface. The discrete Laplacian of a vertex $\hat{Y}_{i}$ is denoted as:

3 $$\begin{aligned} \delta _{\hat{Y}_{i}} = \frac{1}{|S(\hat{Y}_{i})|} \sum _{j\in S(\hat{Y}_{i})} (\hat{Y}_{i} - \hat{Y}_{j}), \end{aligned}$$

where $\hat{Y}_{j}$ is a neighbouring vertex of $\hat{Y}_{i}$ and $S(\hat{Y}_{i})$ denotes the set of all such neighbours. The discrete Laplacian loss is then given by:

4 $$\begin{aligned} \mathcal {L}_{Laplacian} = \frac{1}{N} \sum _{i=1}^{N} \left\Vert { \delta _{V_{i}} - \delta _{\hat{Y}_{i}} }\right\Vert _{2}^{2}, \end{aligned}$$

where $\delta _{V_{i}}$ and $\delta _{\hat{Y}_{i}}$ are the discrete Laplacian before and after the deformation, respectively.

In our experiments, we used λ=0.1 to balance the weights of the two losses. Implementation and training details are shown in Sect. Methods of Online Resource 1.

### Clinical datasets

We evaluated the Deep-Motion-Net framework on data from four liver cancer patients. Each dataset included: 1) a 4D CT with 10 phases (10×3D volumes, $512\times 512\times 105$, $0.98\times 0.98\times 2.0$ mm$^3$ resolution) and 2) two kV scan series (4–5 min free-breathing, full gantry rotation) acquired at the start of treatment on different days. In line with clinical practice, the kV scans were liver-centred with a constrained FOV, so some projection angles captured only the partial liver.

### Synthetic dataset generation

Model training requires paired organ motion instances and corresponding kV images, which cannot be directly acquired. We therefore generate synthetic data for both training and evaluation. Patient-specific motion patterns are extracted from 4D CT images, and new instances are generated by interpolating and modestly extrapolating these motions. The process is as follows: 1) 4D CT images are analysed using the SuPReMo toolkit [20], which produces motion models linked to surrogate signals; 2) new motion instances are generated by perturbing these signals; 3) the resulting motion fields deform the reference (phase 0) CT volume; 4) digitally reconstructed radiographs (DRRs) are generated from the deformed volumes at all required projection angles, and 5) the DRRs are style-transferred to match kV image intensity and noise characteristics. This yields realistic synthetic kV images with known ground-truth 3D motion states. Finally, the target organ is segmented from the reference CT, and a template mesh is constructed. Ground-truth node positions are obtained by interpolating the displacement field at mesh nodes.

For each patient, we generated 550 deformation states, divided into training (350), validation (100), and test (100) sets. For training and validation states, 100 uniformly sampled synthetic kV images were generated at projection angle intervals of $3.6^\circ$ (yielding 35,000 training and 10,000 validation images). For test states, 50 kV images were generated at *randomly* sampled projection angles (giving 5,000 test images). To ensure proper FOV alignment between synthetic and real kV images, each synthetic image was generated using FOV origin header information from a corresponding real image (acquired at a comparable projection angle) within one of the scan series. The real kV images played no further role in the experiment. Model-predicted and ground-truth 3D liver shapes were compared for all synthetic kV images in the test sets. Full details are provided in Sect. Introduction of the Supplementary Material (Online Resource 1).

## Results

### Experiments on synthetic data

**Fig. 3:**
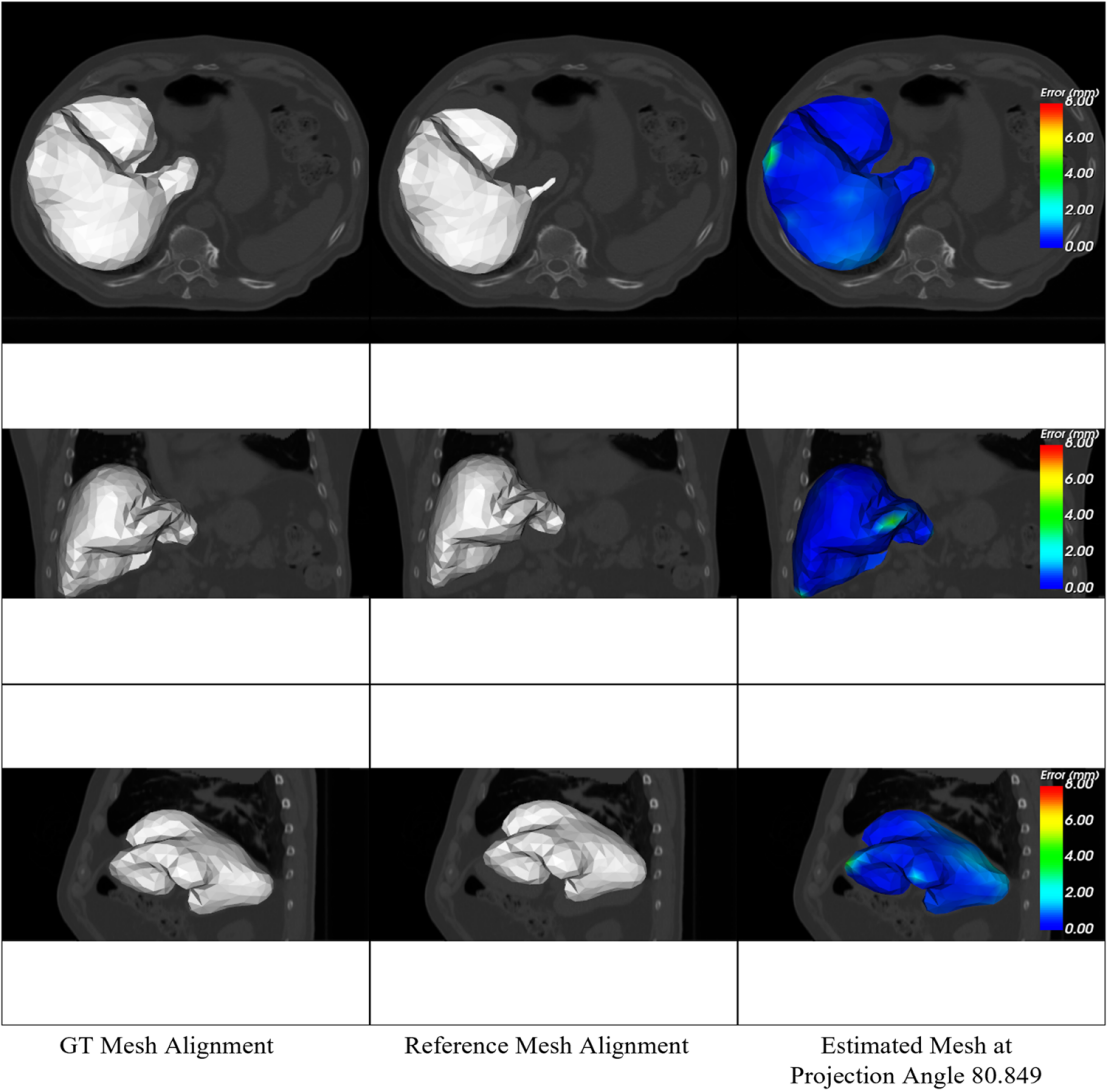
Visualisations of ground-truth deformed (left), template (middle), and estimated deformed (right) 3D liver shapes overlaid on the deformed 3D CT volume. Rows 1-3 show axial, coronal, and sagittal views, respectively. Results are for the *worst* performing test case for patient 1: projection angle $80.849^\circ$ and deformation state with the highest errors. Contours in the right column show the spatial error distribution on the surface. Results for patients 2–4 are in Sect. Discussion of Online Resource 1

We first evaluated the framework on synthetically generated motion states with known ground-truth deformations. Summary results are presented in Table 1. The study employed Euclidean distance as the metric to evaluate the distance errors between ground-truth and estimated shapes. Distributions of mean and peak errors for each test set, and for each projection angle (divided into bins), are shown in Fig. 2. Finally, surface renderings of the ground-truth, reference, and predicted mesh shapes, overlaid on deformed 3D CT volumes, are presented in Fig. 3.

For each of the four test sets, the overall mean error was low: ≤0.22 mm. Within each test set, the maximum peak errors (i.e. overall max error found in any of the deformation states and at any node) were rather higher, ranging from 4.36 mm for patient 4 to 14.66 mm for patient 3. These peak values occurred for the deformation states with the highest ground-truth displacement (namely: 28.12, 34.91, 49.63, and 25.64 mm for patients 1, 2, 3, and 4, respectively) for each test set. Higher underlying ground-truth displacements corresponded, *in general*, with higher peak errors, although mean errors were consistently low. As indicated by Fig. 2, the displacement prediction accuracy was almost independent of the image projection angle: box-and-whisker plots of both mean and peak errors are very similar across the range of angles.

Finally, it is important to note that the higher peak errors in all cases were extremely localised within the meshes. This is indicated firstly by the low overall mean values and more so by the $99^\textrm{th}$ percentile errors (Table 1), which were below 1 mm for 3/4 test sets and below 2 mm for the fourth. That is, the errors were low, even sub-millimetres, for the vast majority of the mesh nodes. The point is further illustrated by the renderings of predicted mesh shapes colour-mapped by displacement error in the right columns of Fig. 3. For liver meshes, the error was close to zero over most of the surface and had only very localised regions of higher values.

While the overall mean prediction errors were consistently low (Table 1), the maximum peak errors warrant detailed spatial analysis to substantiate the claim of extreme localization. Table 2 presents a quantitative breakdown of the percentage of mesh nodes exceeding specific error thresholds across all test instances for each patient.

The analysis confirms that prediction errors are indeed extremely localised. For all patients, fewer than 3.5% of nodes exceed a 5 mm error threshold, which is well within the clinical SBRT margin. Even for Patient 3, who exhibited the highest error burden, only 1.42% of nodes exceed 10 mm despite a maximum peak error of 14.66 mm. This quantitatively validates that large errors are rare outliers affecting a very small fraction of the anatomical geometry.

As shown in Table 2, high-error regions exhibit anatomically consistent patterns across patients. Peak errors predominantly occur at the superior or postero-superior liver dome—the region undergoing the largest displacement during respiratory motion and most susceptible to sliding motion artefacts at the liver–diaphragm interface. For Patient 3, the postero-superior dome exhibited mesh–image misalignment even in the SuPReMo-generated ground truth due to motion artefacts in the planning 4D CT, particularly at the initial and final time points of large-amplitude deformations. This indicates that the reported maximum error reflects a mismatch between the model’s physiologically smooth prediction and fragmented reference geometry containing reconstruction artefacts.

Peak errors are strongly correlated with respiratory phase rather than projection angle. The maximum errors occur predominantly at end-inspiration and end-expiration phases, corresponding to the largest displacements of the liver dome and the extremes of the respiratory cycle, where 4D CT phase-binning artefacts are most severe. Conversely, as demonstrated in Fig. 2, prediction errors remain largely independent of projection angle. The model maintains consistent sub-millimetre mean accuracy across the full $360^{\circ }$ gantry rotation. This confirms that the Deep-Motion-Net successfully learns 3D anatomical representations from kV X-ray images and that localised high errors result from internal geometric inconsistencies in the 4D CT ground truth rather than angle-dependent feature extraction failures.

Additional statistical characterisation of model performance, including inter-patient variability analysis, displacement–error correlation analysis, and CT quality impact assessment, is provided in Section 6 of Online Resource 1.

**Table 2:** Quantitative spatial distribution of reconstruction errors across the tetrahedral mesh nodes for each patient case. Values represent the percentage of total nodes falling within specific error thresholds across all test instances

Patient	Nodes with error < 2 mm	Nodes with error 2–5 mm	Nodes with error 5–10 mm	Nodes with error > 10 mm	Anatomical location of peak
1	98.42%	1.51%	0.07%	0.00%	Superior Dome
2	97.45%	2.22%	0.33%	0.00%	Inferior Edge
3	**94.28%**	**3.12%**	**1.18%**	**1.42%**	**Postero-superior Dome**
4	99.14%	0.81%	0.05%	0.00%	Posterior Border

### Evaluation on real kV images

Our second set of experiments used real in-treatment kV images from each patient’s second scan series (i.e. the series *not* used during training data creation). These series contained 1378, 1310, 1320, and 1292 images for patients 1, 2, 3, and 4, respectively. In the absence of ground-truth 3D deformations, direct assessment of prediction errors is impossible. Therefore, two approaches were adopted: 1) semi-quantitative assessment based on an image similarity metric between input real kV images and model-generated DRRs and 2) qualitative assessment based on overlaying model-predicted liver boundaries on input kV images. For the qualitative assessment, all images in the scan series were used. To reduce computation time (associated, in particular, with spline deformation of the image volumes), only 100 images, uniformly sampled, were used from each patients’ series in the similarity-based assessment.

#### Mutual information-based assessment

For each real kV image, the predicted 3D deformation was applied to the reference CT using a thin-plate spline transformation, followed by DRR generation at the corresponding projection angle. MI was computed within a liver-specific region of interest to ensure the comparison was restricted to anatomically relevant areas. The same procedure was repeated using the undeformed CT volume as a baseline.

Table 3 summarises the results. Across all patients, DRRs generated from motion-corrected CT volumes consistently achieved higher MI scores than those generated from the reference CT, indicating improved correspondence with real kV images. One-way ANOVA tests confirmed that these improvements were statistically significant for all cases (p = 0.0449, 0.0283, 0.0453, and 0.0423 for patients 1–4, respectively; α = 0.05).

**Table 3:** Mutual information (MI) similarity scores between real kV images and digitally reconstructed radiographs (DRRs) generated at identical projection angles for each patient case. Values are reported as *mean (standard deviation)*, computed from 100 images sampled from each scan series. Column 2 presents results obtained using the reference (undeformed) CT volume, while Column 3 presents results obtained using the CT volume deformed by the model-predicted deformation fields. All values were computed within the liver region

Case	Reference	Deformed
1	1.16±0.31	1.33±0.23
2	1.14±0.21	1.39±0.14
3	1.13±0.27	1.28±0.23
4	1.31±0.25	1.47±0.15

#### Qualitative assessment by boundary overlay

kV images from the mentioned scan series were fed into the trained models to obtain 3D mesh shape predictions. All images in the second scan series were used. From each predicted mesh, a corresponding binary image volume was generated. Finally, the projected liver surface boundaries were obtained through ray-tracing on these binary image volumes and superimposed on the respective input kV images. Further details are provided in Supplementary Sect. Results (Online Resource 1), with full 2D animations in Online Resources 2 and 3.

### Comparison model

We compared our method with IGCN [9, 10], which predicts 3D organ shapes from single-view X-rays but is limited to fixed projection angles and surface-only geometries. For fairness, both models were trained and evaluated under these constraints. IGCN was trained on surface meshes extracted from our ground-truth volumes using synthetic 0$^{\circ }$ kV images (256 × 256 resolution, batch size 1, learning rate 0.0001, 1000 epochs). At test time, 0$^{\circ }$ images were input to IGCN to predict surface meshes, while our model’s volumetric outputs were converted to surfaces for direct comparison.

Table 4 shows our method achieved higher accuracy in liver surface deformation across all patient cases. One-way ANOVA tests confirmed statistical significance: for each test set, we calculated mean Euclidean distances between ground-truth and predicted surface meshes, producing two groups of 100 mean error values (one per model, corresponding to 100 deformed states in the test set). The resulting p values of 0.0419, 0.0088, 0.0074, and 0.0485 for patients 1–4, respectively, confirmed significance at α=0.05.

**Table 4:** Summary prediction error statistics comparing our model and IGCN. All errors are computed from predicted organ surface meshes. Patient case numbers are in column 1. $E_{Pred}^{Ours}$ and $E_{Pred}^{IGCN}$ denote prediction errors for our method and IGCN, respectively. $U_{GT}$ denotes ground-truth deformation magnitudes. *Mean (std)*: means (standard deviations) across all nodes, deformation states, and projection angles. *Mean peak*: means of peak values per deformation state across all projection angles. *Max peak*: overall maximum values across all nodes, states, and angles. 99$^{th}$ Percentile: $99^\textrm{th}$ percentile values across all nodes, states, and angles. All values in mm

Case		Mean (std)	Mean peak	Max peak	$99^\textrm{th}$ Percentile
1	$E_{Pred}^{Ours}$	0.17±0.11	0.89	4.91	0.47
	$E_{Pred}^{IGCN}$	0.18±0.25	1.13	6.37	0.93
	$U_{GT}$	10.11±1.24	13.94	28.12	13.53
2	$E_{Pred}^{Ours}$	0.14±0.15	1.53	7.13	0.67
	$E_{Pred}^{IGCN}$	0.17±0.21	2.81	8.79	1.18
	$U_{GT}$	11.37±1.55	15.41	34.91	14.09
3	$E_{Pred}^{Ours}$	0.15±0.13	1.46	13.83	0.77
	$E_{Pred}^{IGCN}$	0.19±0.23	2.05	14.31	1.23
	$U_{GT}$	14.52±2.39	18.71	49.63	16.74
4	$E_{Pred}^{Ours}$	0.12±0.09	0.78	3.98	0.44
	$E_{Pred}^{IGCN}$	0.14±0.17	0.97	5.31	0.77
	$U_{GT}$	10.01±1.05	12.17	25.64	11.33

## Discussion

We presented a CNN-GNN–based model for recovering 3D volumetric organ mesh deformations from single in-treatment kV X-ray images at arbitrary projection angles. The framework is attractive as it requires only standard imaging during radiotherapy, without MRI, surrogate signals, or invasive fiducial markers. To our knowledge, this is the first deep learning framework able to reconstruct volumetric 3D organ models from arbitrary single-view images across full treatment scan series.

Training relied on synthetic data from the SuPReMo toolkit, necessary due to the absence of ground-truth deformations for real kV images. Performance therefore depends on the fidelity of synthetic motions derived from 4D CT breathing cycles, which model respiratory motion as the dominant deformation source rather than the full variability introduced by other physiological factors such as abdominal pressure changes or deformation caused by nearby organs. We approximated this by generating bounded random motion states, but future work will incorporate more realistic variability from extended kV sequences. Although developed for respiratory motion, the method can generalise to other organ motions with suitable training data, as it imposes no assumptions of periodicity or motion type.

Results showed robust accuracy; however, prediction errors were influenced by CT volume quality: patients with motion-related reconstruction artefacts exhibited higher peak errors, though these remained localised, and mean errors stayed low. For real kV images without ground truth, qualitative overlays showed consistent, smooth liver contours across frames, and deforming reference CTs improved image similarity, suggesting sensible predictions.

Qualitative assessment of real kV images showed consistent, smoothly transitioning predicted organ contours between frames without noticeable jumps, despite independent frame predictions. This suggests robustness to noise and irregularities in real images. It is important to note that drawing such boundaries poses inherent challenges for clinicians or radiologists, given the nature of these low-contrast kV X-ray images. Building on these findings, future work will aim to systematically quantify the model’s robustness across a broad range of FOV variations within the same patient, thereby further assessing its adaptability and reliability across diverse clinical configurations.

A key limitation of the present study is that the training data are derived from the SuPReMo motion model, which is itself constructed from phase-averaged 4D CT and therefore does not explicitly capture irregular respiratory behaviour such as abrupt intra-cycle variations, coughing, or strong patient-specific breathing patterns. While surrogate signal perturbations were introduced to increase variability, these represent a pragmatic approximation rather than a physiologically comprehensive model. As a result, the reported performance is interpreted within the context of typical respiratory motion, and future work will focus on incorporating more realistic, temporally resolved motion data to further assess robustness under irregular breathing conditions.

Beyond motion modelling, additional limitations include the current focus on a single target organ, ignoring OARs and surrounding anatomy. Extending the model to multiple meshes is feasible but may require constraints to prevent overlap or larger unified meshes. Moreover, the current predictions are purely image-driven. Nevertheless, the volumetric formulation of Deep-Motion-Net provides a natural foundation for future integration of biomechanical constraints, such as constitutive models implemented through finite element method–based formulations, to further enhance the physical plausibility of the predicted deformations. From a clinical perspective, the primary aim of this work is to improve the therapeutic index by reducing normal tissue complication probability NTCP while maintaining or increasing tumour control probability TCP, thereby enabling higher radiation doses without the need for invasive tracking. In this context, the inter-fraction and intra-fraction adaptation workflows described in this study (Online Resource 1) are intended to illustrate potential clinical integration pathways enabled by the proposed motion estimation framework. While the present work validates the accuracy and performance of the motion prediction component, full end-to-end validation, including dosimetric verification, system latency characterisation, and failure detection and recovery strategies, remains beyond the scope of this study and is the subject of ongoing and future work. Accordingly, the proposed workflows should be interpreted as enabling concepts rather than fully deployed clinical solutions.

## Supplementary Information

Below is the link to the electronic supplementary material.

Supplementary file 1 (pdf 6424 KB)

Supplementary file 2 (docx 69456 KB)

Supplementary file 3 (docx 64941 KB)
