# Supplementary material for: Deep-Motion-Net: GNN-based volumetric liver shape reconstruction from single-view 2D projections
Source: Int J Comput Assist Radiol Surg. 2026 May 13;21(6):1253–62. doi: 10.1007/s11548-026-03685-1 (PMC13451316; doi:10.1007/s11548-026-03685-1)
Supplement: Supplementary file 1 [file 11548_2026_3685_MOESM1_ESM.pdf]

Online Resource 1 (Supplementary): Deep-Motion-Net: GNN-based volumetric organ shape reconstruction from single-view 2D projections

Article Title	Deep-Motion-Net: GNN-based volumetric organ shape reconstruction from single-view 2D projections
Conference Name	International Conference on Information Processing in Computer-Assisted Interventions
Journal Name	International Journal of Computer Assisted Radiology and Surgery
Affiliation	University of Leeds
Email Address	isurusuranga.wijesinghe@gmail.com

1 Synthetic dataset generation

Model training requires paired sets of organ motion instances and corresponding kV images. However, to the best of our knowledge, there are no means of directly measuring such motions while also acquiring the requisite images. Hence, we use synthetically generated data to train and, in this work, to evaluate the model. Plausible patient-specific motion patterns are extracted from 4D-CT images, and new synthetic instances are produced by interpolating and, within reasonable bounds, extrapolating from these. The process is as follows: 1) 4D-CT images are analysed using the SuPReMo toolkit, which produces, among other things, a model of the motion present in the images, linked with appropriate surrogate signals; 2) new, yet plausible motion instances are generated from this model by randomly perturbing the surrogate signal; 3) the resulting motion fields are used to deform the reference (phase 0) CT volume; 4) Digitally Reconstructed Radiographs (DRRs) are generated from these deformed volumes for all required projection angles, and 5) the DRRs are style transferred to match kV image intensity and noise distributions. The result is a set of realistic 'kV' images of the deformed anatomy acquired at various projection angles, for which ground-truth 3D motion states are known. Finally, the target organ is segmented from the reference CT volume, and an organ template mesh is constructed. Full details are presented in the following sections.

1.1 Generation of synthetic motion states from 4D-CT data

SuPReMo is a toolkit for simultaneously estimating a motion model and constructing motion-compensated images from a 4D-CT dataset. The resulting model describes the anatomical motion present in the raw data over a single (averaged) breath cycle and is linked with corresponding scalar surrogate signals derived, for example, from breathing traces or image features. For formulation reasons (see [1, 2]), two surrogate signals are required; however, these needn't be independent, and as a practical measure in each case we constructed the second signals as temporal derivatives of the first, computed for example using finite differences. Our 4D-CT datasets each decompose the breath cycle into 10 bins (phases).

First, we fit SuPreMo's motion model for each individual case with input surrogate signal and 4D-CT data. This returns the motion-compensated reconstructed 3D-CT (MCR) image volume and the fitted motion model, which are then utilised to simulate new motion states by varying the input surrogate signal. Each point s_i, $i \in [0, 9]$ on the curve is randomly perturbed by a value in the range $\pm 0.4 s_i$. Extrapolating beyond this range is more likely to cause unrealistic motion and even folding in the resulting images. A new surrogate signal is then created by fitting a 3rd order polynomial to the new points. The latter ensures the new signal remains smooth over the full breath cycle. Using this new signal, and MCR as a reference volume, SuPReMo's motion model generates corresponding deformed 3D-CT volumes and their related deformation vector fields.

For each patient case, we created 11 separate surrogate signals, each comprising ten deformed configurations, resulting in 110 synthetic deformation states in total. To introduce more diversity into the synthetic motion instances and substantially deviate from the original 4D-CT data, we incorporated rigid motions by applying random translations/shifts along the left-right (LR), anterior-posterior (AP), and superior-inferior (SI) directions and produced a total of 550 deformed states. This is also advantageous during testing on real in-treatment kV images, as it reflects the variations in onboard patient setup across different scan series or fractions, potentially leading to shifts in the in-treatment kV images.

1.2 Generation of synthetic kV X-ray images

For each deformation state, DRRs were obtained from the deformed 3D-CT volumes using the Siddon-Jacobs ray tracing technique (itk-rtk version=2.4.0) ([3, 4]). The latter, given a source position and projection direction, computes the line integral of Hounsfield unit densities along ray lines to a prescribed 2D plane. In radiotherapy, in-treatment kV images are acquired perpendicularly to the anatomical axial direction.

The resulting DRRs lack scatter properties and noise characteristics of genuine kV X-ray images, making them appear sharper and higher in contrast. We, therefore, used a CycleGAN ([5]) by conditioning on the projection angle to effect the DRR-to-kV style transformation. The conditional CycleGAN was trained on an unpaired set of DRRs and real in-treatment kV images for each case separately since the FOV acquisition varies from patient to patient. Histogram equalisation was first applied on the kV images to stretch the intensity histograms concentrated on a narrow interval to

a broader range and increase contrast. All DRRs were then passed to the conditional CycleGAN to create the final synthetic kV X-ray images. We initially used transposed convolution layers for the decoder part of the generators; however, we encountered checkerboard-like artefacts in several cases. These were eliminated by instead using convolution layers followed by pixel shuffle layers ([6]).

1.3 Creation of template meshes

Binary masks of the relevant organs were extracted from reference 3D-CT volumes using 3D Slicer's Segment Editor. These masks were then used to generate tetrahedral meshes using the Iso2Mesh (version=1.9.6)([7]) tool in MATLAB (version=R2019a). The resulting meshes were scaled and translated to reflect image voxel sizes and correct image origins. By these means, the meshes are physically aligned with the relevant anatomical regions in the 3D-CT volumes. Ground-truth positions of the mesh nodes for each of the generated deformation states are produced by interpolating the displacement vector field at the node positions.

2 Implementation and training details

The network was implemented using PyTorch (version=1.7.1) and PyTorch-geometric (version=1.6.3) [8]. Training employed the Adam optimizer with learning rate of 0.0002, weight decay of 0.001, and batch size 16 for both training and validation. Group normalisation normalised hidden layer outputs in both 2D-CNN and GNN. Single-headed attention was used in GAT layers due to memory restrictions. Early stopping monitored validation loss with 30-epoch patience; learning rate decreased by factor of 0.8 after eight consecutive epochs without improvement. Weights were initialised per [9]. Gradient descent converged after 400 epochs. Optimal network components were determined through experiments described in 5. Training took approximately 36 hours at 256×256 resolution on a Nvidia Quadro RTX 4000 GPU using a Precision 7820 Tower XCTO Base workstation.

3 Experiments

Our second set of experiments used real in-treatment kV images from each patients' second scan series (i.e. the series *not* used during training data creation). These series contained 1378, 1310, 1320, and 1292 images for patients 1, 2, 3, and 4, respectively. In the absence of ground-truth deformations, direct assessment of prediction errors is impossible. Therefore, we performed a qualitative assessment (visual inspection) based on overlaying model-predicted liver boundaries on input kV images. For the qualitative assessment, all images in the scan series were used.

Histogram-equalised kV images from the mentioned scan series were fed into the trained models to obtain 3D mesh shape predictions. As mentioned, all images in the scan series were used. From each predicted mesh, a corresponding binary image volume was generated. Finally, the projected liver surface boundaries were obtained through ray-tracing on these binary image volumes, and superimposed on the respective input kV images. Samples from each patient are shown in Figure 1. Online Resources 2 and

3, moreover, include animated versions based on the full scan series of each patient case for further reference. These latter most effectively show (qualitatively) the consistent alignment of the model predictions with the input images across many breathing cycles and the full rotation of the treatment gantry.

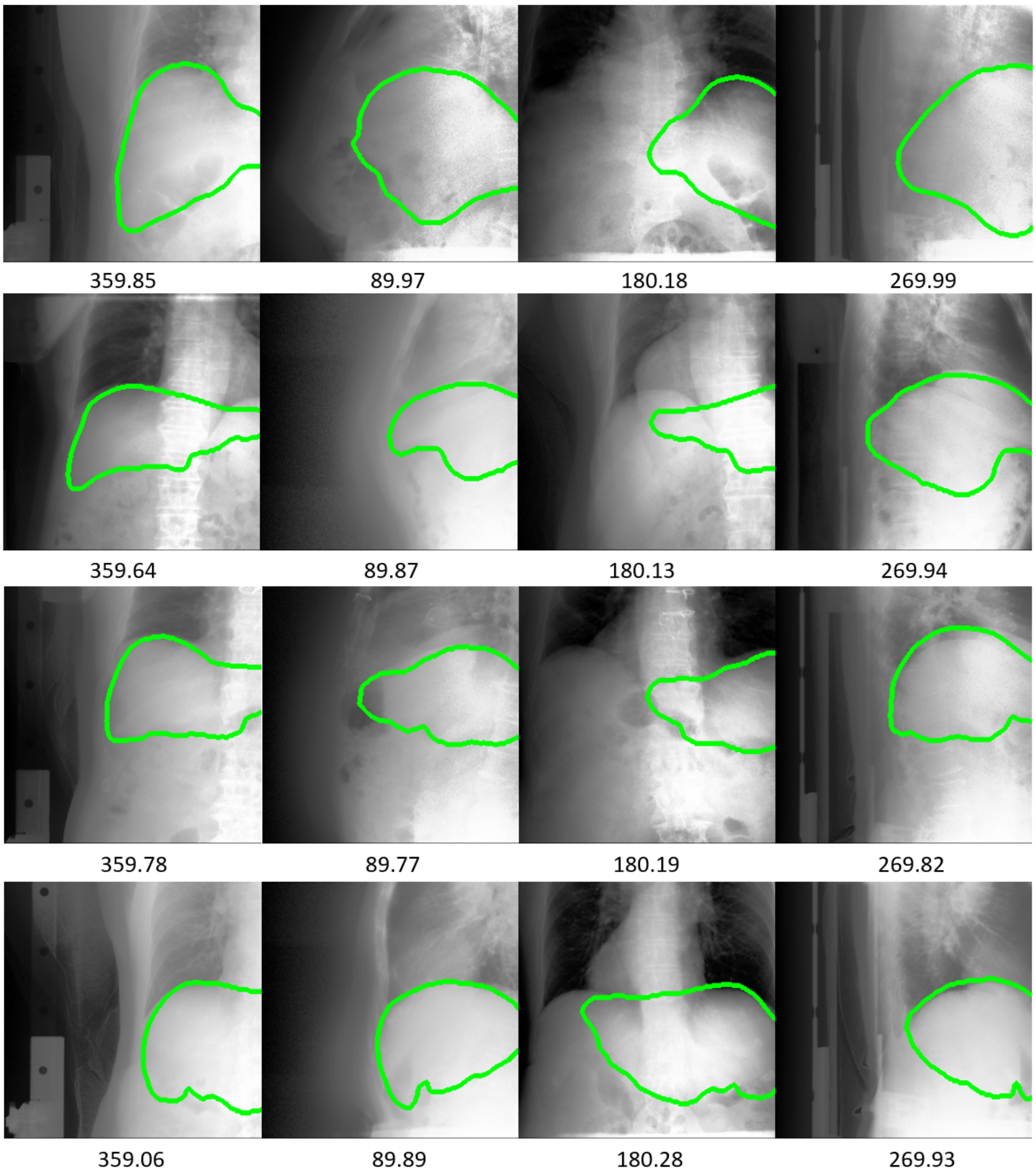

Fig. 1 Samples of overlaid predicted liver boundary projections on corresponding real kV images for the four patients. Rows 1-4 show, respectively, results for patients 1-4. Results for images acquired at four projection angles (degrees, indicated below the images) are shown.

4 Results

4.1 Patient 1

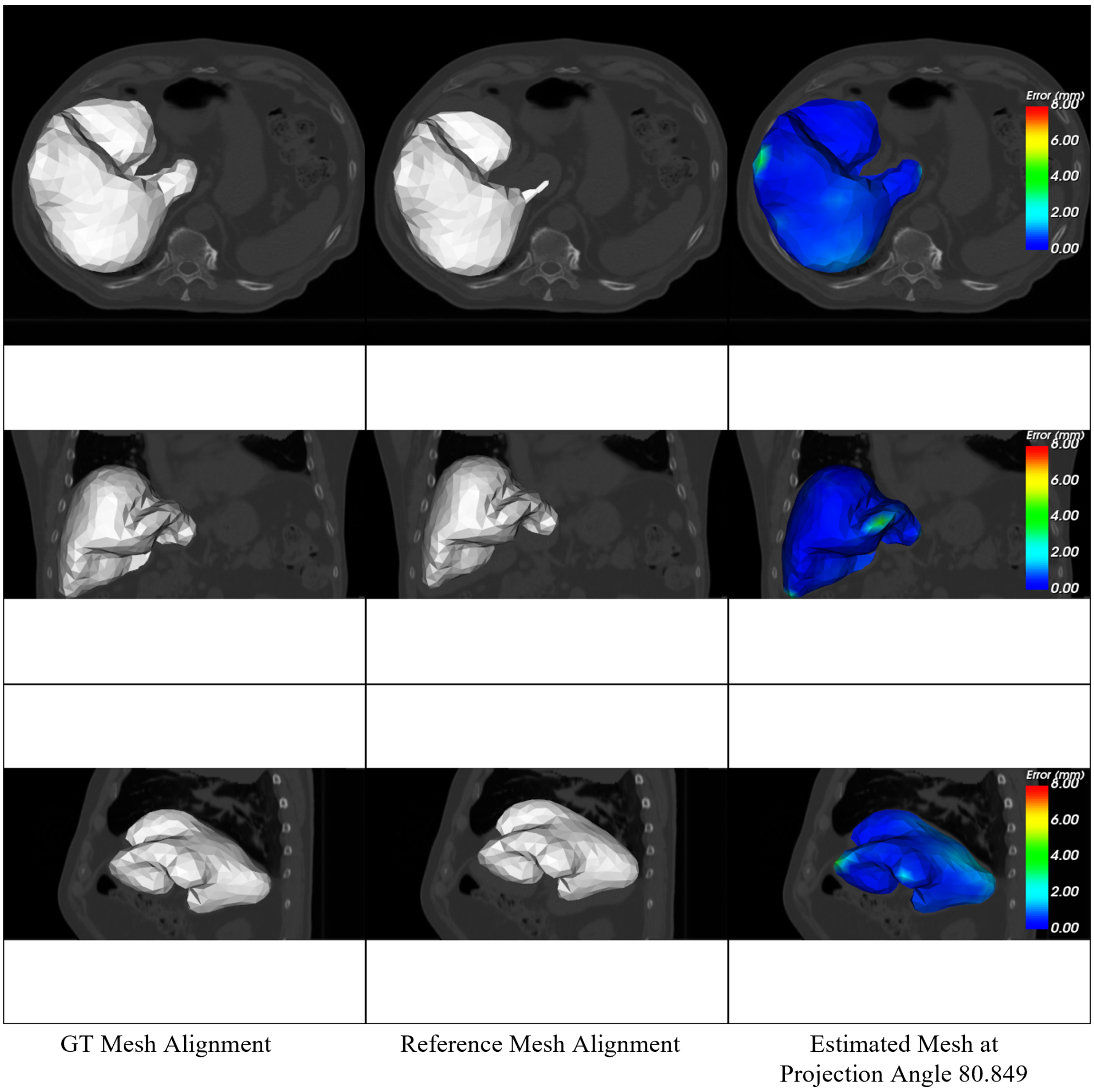

Fig. 2 Visualisations of ground-truth deformed (left column), template (middle column), and estimated deformed (right column) 3D liver shapes. Meshes are overlaid on the deformed 3D-CT volume. Rows 1-3 show, respectively, axial, coronal, and sagittal views. Results are shown for the *worst* performing test case for patient 1: image projection angle 80.849°, and deformation state producing the highest errors. Contours in the right column indicate the spatial distribution of errors on the surface.

4.2 Patient 2

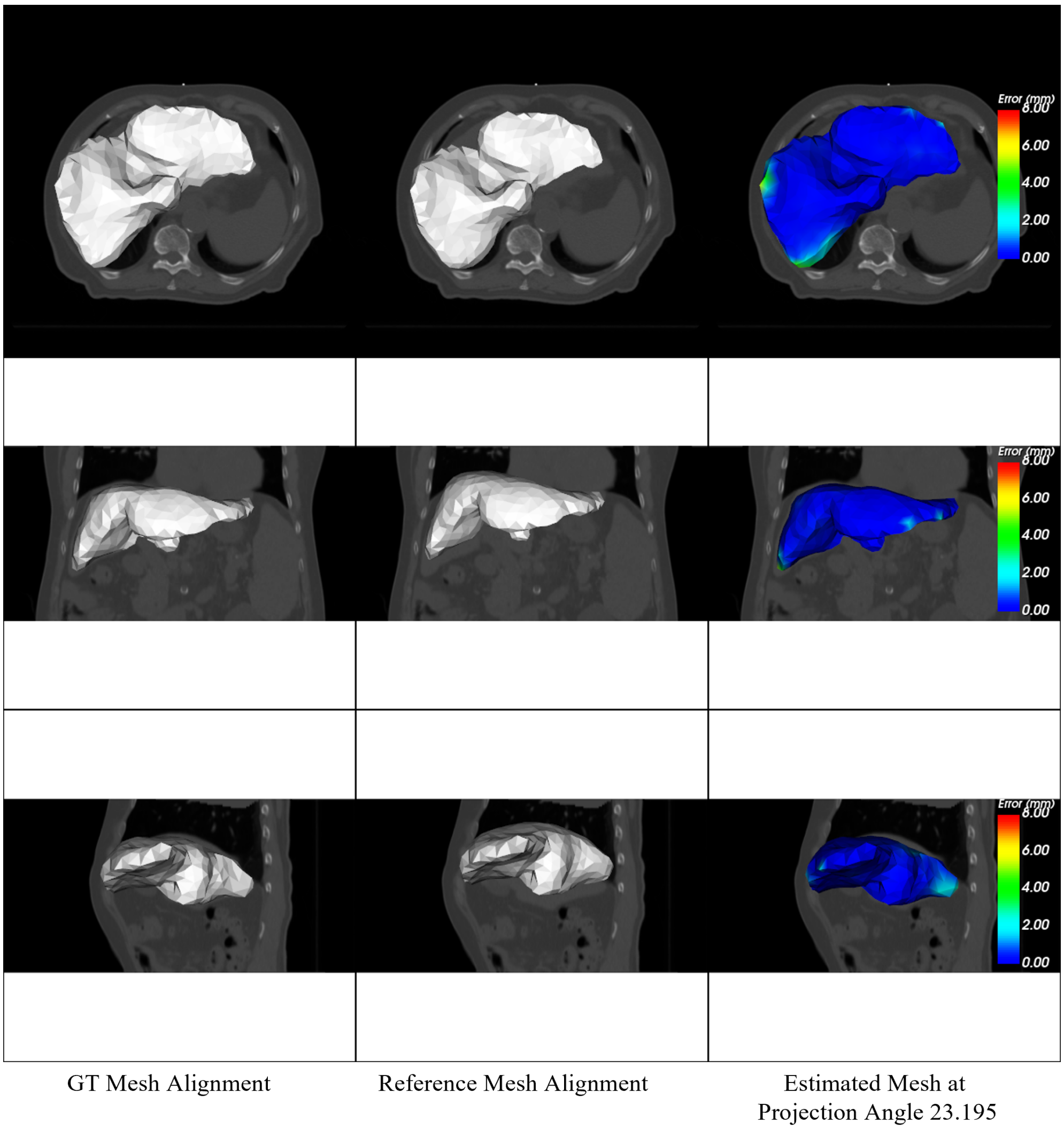

Fig. 3 Visualisations of ground-truth deformed (left column), template (middle column), and estimated deformed (right column) 3D liver shapes. Meshes are overlaid on the deformed 3D-CT volume. Rows 1-3 show, respectively, axial, coronal, and sagittal views. Results are shown for the *worst* performing test case for patient 2: image projection angle 23.195°, and deformation state producing the highest errors. Contours in the right column indicate the spatial distribution of errors on the surface.

4.3 Patient 3

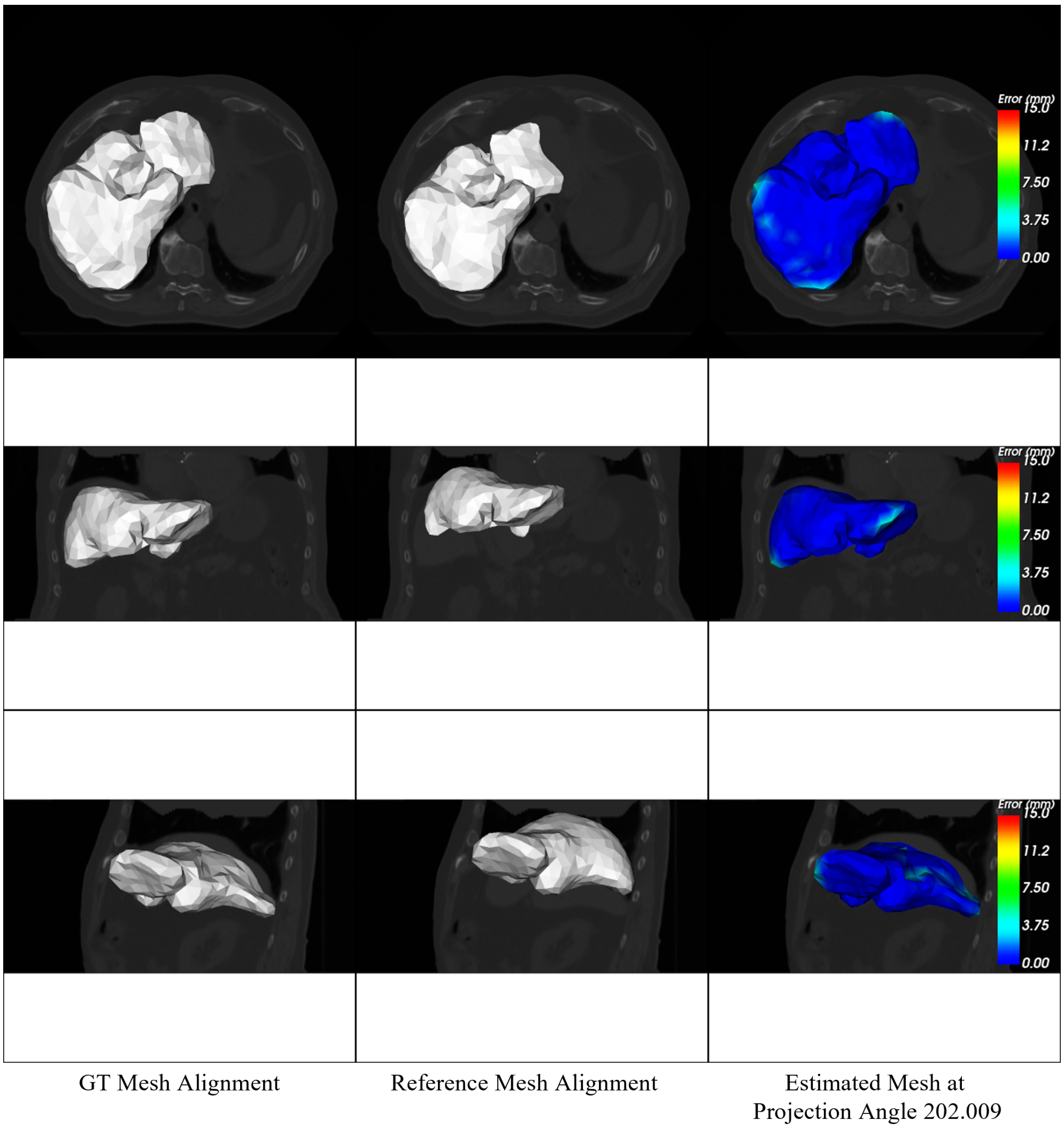

Fig. 4 Visualisations of ground-truth deformed (left column), template (middle column), and estimated deformed (right column) 3D liver shapes. Meshes are overlaid on the deformed 3D-CT volume. Rows 1-3 show, respectively, axial, coronal, and sagittal views. Results are shown for the *worst* performing test case for patient 3: image projection angle 202.009°, and deformation state producing the highest errors. Contours in the right column indicate the spatial distribution of errors on the surface.

4.4 Patient 4

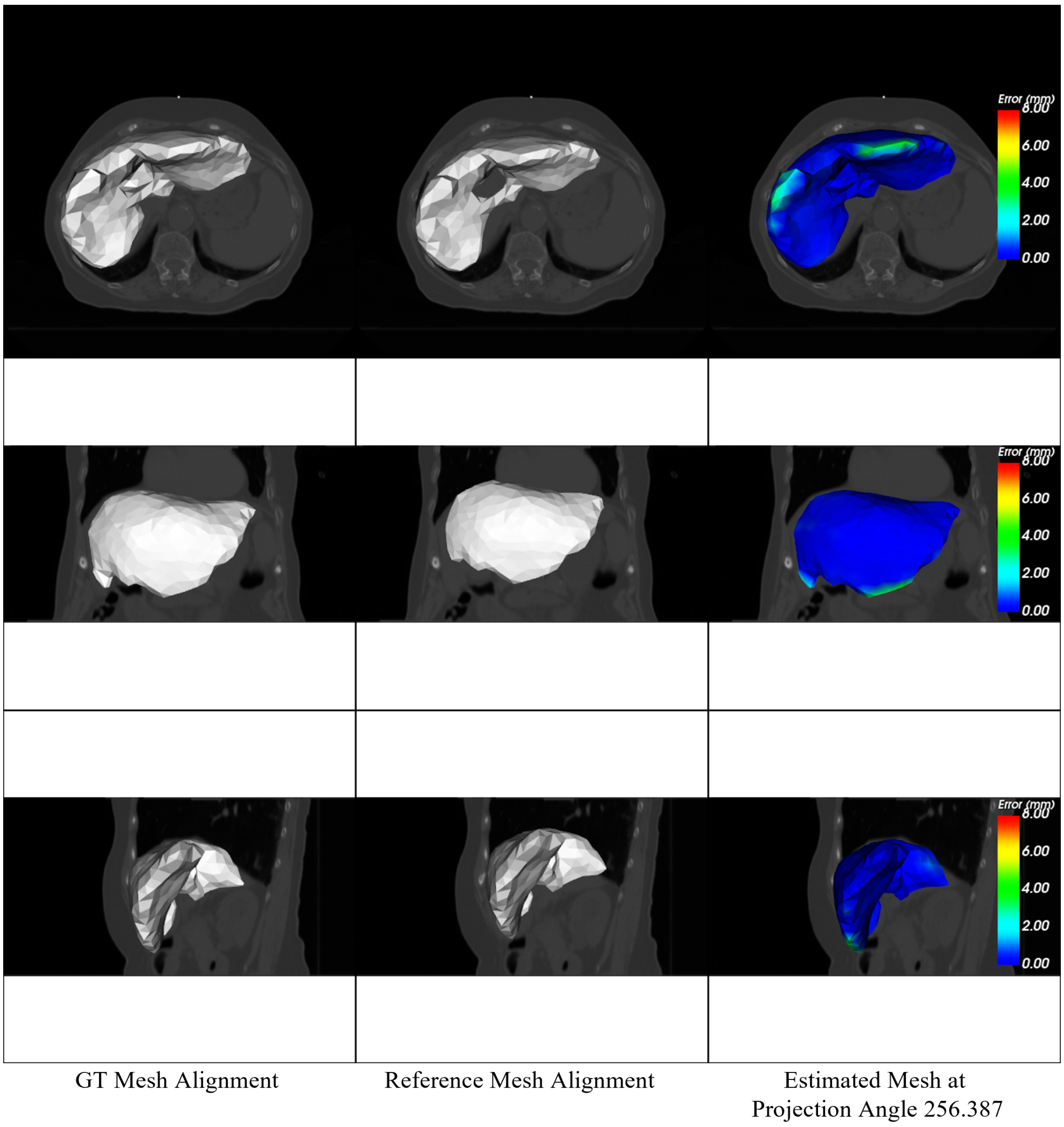

Fig. 5 Visualisations of ground-truth deformed (left column), template (middle column), and estimated deformed (right column) 3D liver shapes. Meshes are overlaid on the deformed 3D-CT volume. Rows 1-3 show, respectively, axial, coronal, and sagittal views. Results are shown for the *worst* performing test case for patient 4: image projection angle 256.387°, and deformation state producing the highest errors. Contours in the right column indicate the spatial distribution of errors on the surface.

5 Ablation study

Table 1 Impact of reducing the number of image encoder MLPs. All values reported in mm.

Experiment	Mean (std)	Mean peak	Max peak	99th Percentile
1	0.21±0.18	1.92	7.53	1.19
2	0.23±0.22	2.88	8.47	1.45

This section describes the experiments we performed to determine the impact of each model component on the overall model performance. All experiments were conducted by training the model for 400 epochs. The synthetic data generated for patient 1 were used; hence, all results should be compared with patient 1.

We first assessed the impact of the four MLPs in the image encoder. As described, our incorporated an MLP for each convolutional layer, with each MLP responsible for five features per vertex, for a total of 20 features. Two experiments were conducted: 1) we removed MLPs associated with the first two convolutional layers, resulting in only ten image features per vertex; 2) we removed all MLPs except the final one, associated with the final convolutional layer, resulting in five features per vertex. In each of these cases, projection angle information was integrated into the input layer. Results are shown in Table 1, which demonstrate the importance of all four MLPs.

Table 2 Impact of removing gantry (projection angle) information from the image encoder. All values reported in mm.

Mean (std)	Mean peak	Max peak	99th Percentile
0.23±0.21	2.81	9.33	1.27

We next explored the impact of projection angle information in the image encoder by omitting this information from the input layer. For this experiment, the number (i.e. four) of MLPs remained constant. The results in Table 2 show that feeding angle information to the input layer is more successful.

Table 3 Impact of removing skip connections from the mesh deformation network. All values reported in mm.

Mean (std)	Mean peak	Max peak error	99th Percentile
0.20±0.19	2.04	7.69	1.31

In the next experiment, we focused on the effect of residual connections used in the mesh deformation network by simply removing them. Results in Table 3 indicate our approach with residual connections was superior.

Table 4 Impact of replacing graph-attention layers with graph convolutional network layers. All values reported in mm.

Mean (std)	Mean peak	Max peak	99th Percentile
0.18±0.16	1.51	7.05	0.83

Finally, we explored the impact of the graph attention layers in the mesh deformation network by replacing them with GCN layers. The architecture was otherwise unchanged. Results in Table 4 demonstrate that utilizing GAT layers is more effective.

We have extended the ablation study to include Patients 2, 3, and 4 (see Table 5). The results confirm that the architectural benefits are consistent across all cases. Specifically, for Patient 3, who exhibited the highest errors due to 4D-CT artefacts, the current architecture provided the most stable performance compared to simplified alternatives.

Table 5 Cross-patient ablation study for architectural components. Values represent the Mean Error ± Standard Deviation (mm). The 'Proposed' row contains the exact values reported in Table 1 of the manuscript.

Configuration	Patient 1	Patient 2	Patient 3	Patient 4
**Proposed (Full Model)**	**0.16 ± 0.13**	**0.18 ± 0.19**	**0.22 ± 0.34**	**0.12 ± 0.11**
w/o Angle Encoding	0.23 ± 0.21	0.29 ± 0.25	0.50 ± 0.48	0.21 ± 0.18
w/o Residual Connections	0.20 ± 0.19	0.24 ± 0.22	0.38 ± 0.36	0.18 ± 0.15
w/o GAT (using GCN)	0.18 ± 0.16	0.21 ± 0.20	0.31 ± 0.30	0.15 ± 0.13
Reduced FPN (10 features/vertex)	0.21 ± 0.18	0.26 ± 0.23	0.44 ± 0.41	0.20 ± 0.17
Reduced FPN (5 features/vertex)	0.23 ± 0.22	0.28 ± 0.26	0.55 ± 0.51	0.24 ± 0.21

6 Statistical Analysis of Model Performance

This section presents extended statistical analyses characterizing Deep-Motion-Net prediction accuracy across patient cohorts, displacement magnitudes, CT image quality variations, and projection angles.

6.1 Inter-Patient Variability Analysis

To assess whether prediction errors differed significantly across the four patient cohorts, we performed the Kruskal-Wallis H-test.

The test revealed statistically significant differences in prediction errors between patients ($H = 2847.3$, $p < 0.001$), confirming that patient-specific factors substantially influence model performance. To identify which patient pairs exhibited significant differences, we conducted post-hoc pairwise comparisons using Dunn's test with Bonferroni correction for multiple comparisons. The following patterns emerged:

- Patient 3 vs. all others: Significantly higher errors ($p < 0.001$ for all comparisons), consistent with the quantified CT artifact burden reported in Table 2 (28.5% artifact-affected slices, mean gradient sharpness 11.1±3.2)

- Patient 4 vs. Patients 1 and 2: Significantly lower errors ($p < 0.05$), correlating with superior planning CT quality (5.1% artifact-affected slices, mean gradient sharpness 19.3 ± 2.1)
- Patients 1 vs. 2: No significant difference ($p = 0.17$), both exhibiting intermediate CT quality metrics.

These findings demonstrate that the model's prediction accuracy is strongly influenced by the quality of the input planning CT data, with artifact-laden images leading to systematically higher prediction errors.

6.2 Correlation Between Ground-Truth Displacement Magnitude and Prediction Error

Understanding the relationship between displacement magnitude and prediction error is essential for characterizing performance limits and identifying potential failure modes. We performed Spearman rank correlation analysis between nodal ground-truth displacement magnitudes (U_{GT}) and corresponding prediction errors (E_{pred}) across all test instances.

The overall correlation coefficient was $\rho = 0.43$ ($p < 0.001$), indicating a moderate positive correlation. Patient-specific correlations revealed consistent patterns:

- Patient 1: $\rho = 0.38$ ($p < 0.001$)
- Patient 2: $\rho = 0.41$ ($p < 0.001$)
- Patient 3: $\rho = 0.52$ ($p < 0.001$)
- Patient 4: $\rho = 0.35$ ($p < 0.001$)

While prediction error increases with displacement magnitude, the relationship is sub-linear. Importantly, no abrupt failure threshold was observed across the displacement range. Even for extreme displacements exceeding 20 mm, the 99th percentile error remained below 4 mm, indicating that large errors are rare and spatially localized rather than systemic. This suggests the model maintains robustness even when confronted with large-amplitude organ motions.

6.3 Relationship Between CT Image Quality and Prediction Accuracy

Building upon the CT quality metrics introduced in Table 3 of the main manuscript, we performed multivariate regression analysis to quantify the relationship between image quality indicators and prediction accuracy. We fitted a generalized linear mixed model with patient-level random effects, using three image quality predictors:

- Percentage of artifact-affected slices (motion binning artifacts)
- Mean inter-slice intensity difference (discontinuity measure)
- Mean gradient sharpness (blur/artifact severity)

Table 6 presents the regression coefficients, 95% confidence intervals, and p-values for each predictor. All three quality metrics showed statistically significant associations with prediction error. Higher percentages of artifact-affected slices and greater inter-slice intensity differences were associated with increased prediction errors (positive

coefficients), while higher gradient sharpness (indicating sharper, less artifact-affected images) was associated with reduced errors (negative coefficient).

Table 6 Multivariate regression analysis quantifying the relationship between CT image quality indicators and prediction accuracy. All predictors show statistically significant associations with model performance.

**Predictor**	**Coefficient (β)**	**95% CI**	**p-value**
Artifact-affected slices (%)	+0.032	$[0.026, 0.038]$	<0.001
Inter-slice intensity difference	+0.008	$[0.005, 0.011]$	<0.001
Gradient sharpness	−0.015	$[-0.019, -0.011]$	<0.001

The model explains 67.4% of the variance in patient-level mean prediction errors (adjusted $R^2 = 0.674$), demonstrating that CT quality metrics are strong predictors of model performance. The remaining variance (32.6%) likely reflects patient-specific anatomical factors, motion pattern complexity, and stochastic training effects.

6.4 Statistical Characterization of Projection Angle Effects

To move beyond visual inspection of box plots (Figure 2 in the main manuscript) and rigorously test whether prediction accuracy varies systematically with projection angle, we performed both circular-linear correlation analysis and one-way ANOVA across angular bins.

Circular-linear correlation analysis (all patients pooled) yielded $r = 0.03$ ($p = 0.42$), indicating no significant linear relationship between projection angle and error magnitude. To further investigate potential non-linear angle effects, we performed one-way ANOVA across 36 angular bins (10° intervals). The resulting p-values were 0.18, 0.06, 0.08, and 0.21 for patients 1, 2, 3, and 4, respectively. None of the p-values reached significance at $\alpha = 0.05$, confirming that prediction errors do not vary systematically with projection angle.

7 Limitations

The model was trained with synthetic data constructed using the SuPReMo toolkit. Training in this way is essential in the absence of ground-truth deformations corresponding to real kV images; that is, there appears to be no other way of acquiring paired deformation/image sets for this scenario. For similar reasons, in this first study, direct quantitative evaluation of the model performance was also carried out using synthetic data. Naturally, the performance of the model will depend on the fidelity with which real patient motions are reproduced in the synthetic data. While SuPReMo appears to do a reasonable job of characterising the motion present in the input 4D-CT data, these data represent only averaged breathing cycles and do not by themselves provide information about the variability of this motion. Pragmatically, therefore, in the present work, we assumed some bounds on the variations from this average, and randomly generated motion states within these. We are exploring more rigorous

approaches to characterising the patient motion variability (and incorporating this in model training data) based on kV image sequences acquired over several minutes during treatment.

We observed that the model prediction accuracy can be influenced by the quality and characteristics of the CT volumes from which training data are constructed. The higher peak errors found for patients 2 and 3 appear to be at least partly attributable to this. The CT volumes of these two patient cases exhibited certain reconstruction artefacts related to motion. For patient 3, in large amplitude deformed cases, these resulted in some mesh-image misalignment problems even in the ground-truth data (specifically, with the deformed states at initial and final time points) produced from SuPReMo. The deformed images for Patients 1 and 4, by contrast, contained no obvious artefacts or ambiguous anatomy, and peak errors were correspondingly smaller. As emphasised, however, the regions of higher peak errors, even in patients 2 and 3, were nonetheless very localised, and mean errors remained low.

Another limitation of our method, as currently implemented, is that we only estimate the deformation of the target organ; nearby OARs and other anatomy are ignored. However, the model itself imposes no restrictions in this respect. If suitable training data covering the relevant anatomy, and meshes of the relevant anatomical structures can be generated, the model can in principle estimate motions for these in the same way. SuPReMo, for example, provides deformation vector fields for the whole image volume, which could be used for this purpose. OAR and other meshes can normally be created similarly to the liver meshes used here. Potentially, the prediction of deformations for multiple disconnected meshes could result in anomalous overlapping regions. However, such instances would hopefully be rare when the training data include no such behaviour. If needed, separate non-overlap constraints in the formulation could also be conceived, which penalise mesh interpenetration in a way analogous to some contact formulations in computational mechanics. Albeit, this would increase the complexity of the approach. A simpler alternative could be deploying a single mesh covering all relevant anatomy. In all scenarios, it is possible that both model and training data size requirements would increase, though the overall approach would remain the same.

As mentioned, two general approaches to adapting RT treatments could be considered using motion estimates generated by our technique: inter- and intra-fraction adaption. Inter-fraction plan adaption could be achieved by retrospectively estimating the motion that occurred during a treatment fraction and computing motion-compensated spatial distributions of delivered dose (dose accumulation); the treatment plan could then be adapted accordingly for subsequent fractions. No new technology seems to be needed for this, beyond the requirement to image throughout the fraction, rather than during initial patient positioning only. We are currently developing a workflow for this process which, in outline, is as follows: 1) Deep-Motion-Net is trained using patient planning or treatment simulation 4D-CT data; 2) kV images are acquired throughout treatment delivery; 3) Deep-Motion-Net predicts organ motion states for all input kV images; 4) organ (mesh) motion predictions are used to deform patient reference 3D-CT volumes via thin plate spline transformation (with organ nodes as driving control points); 5) positions of the gantry/MLCs at the start and

end of each time period are determined (interpolation in between); 6) dose for the predicted anatomy is computed; 7) dose is mapped to a common reference anatomy (corresponding to reference 3D-CT volume) 8) cumulative delivered dose (sum of contributions from individual motion states) is computed and compared with planned dose.

Intra-fraction adaption requires prediction of target motion, and adjustment of radiation delivery in response, in real-time during the treatment. While our model computes motions quickly (inference time for each input image is ∼27 msec), the inevitable response lag of the treatment system, due both to data processing following a motion input and the physical inertia of the delivery apparatus, means motion predictions must in practice be computed somewhat *ahead of time*. In principle, our approach could be extended to enable this using ideas from sequence-to-sequence learning, e.g. by incorporating RNN (recurrent neural networks) or LSTM (long-short-term memory) components in the model to capture sequences of organ motions, given dynamic sequences of input kV images.

References

[1] McClelland, J.R., Modat, M., Arridge, S., Grimes, H., D'Souza, D., Thomas, D., O'Connell, D., Low, D.A., Kaza, E., Collins, D.J., *et al.*: A generalized framework unifying image registration and respiratory motion models and incorporating image reconstruction, for partial image data or full images. Physics in Medicine & Biology **62**(11), 4273 (2017)

[2] McClelland, J.R., Modat, M., Arridge, S., Grimes, H., D'Souza, D., Thomas, D., O'Connell, D., Low, D.A., Kaza, E., Collins, D.J., et al.: SuPReMo (2017). https://github.com/UCL/SuPReMo

[3] Jacobs, F., Sundermann, E., De Sutter, B., Christiaens, M., Lemahieu, I.: A fast algorithm to calculate the exact radiological path through a pixel or voxel space. Journal of computing and information technology **6**(1), 89–94 (1998)

[4] Siddon, R.L.: Fast calculation of the exact radiological path for a three-dimensional CT array. Medical physics **12**(2), 252–255 (1985)

[5] Zhu, J.-Y., Park, T., Isola, P., Efros, A.A.: Unpaired image-to-image translation using cycle-consistent adversarial networks. In: 2017 IEEE International Conference on Computer Vision (ICCV), pp. 2242–2251 (2017). https://doi.org/10.1109/ICCV.2017.244

[6] Aitken, A., Ledig, C., Theis, L., Caballero, J., Wang, Z., Shi, W.: Checkerboard artifact free sub-pixel convolution: A note on sub-pixel convolution, resize convolution and convolution resize. arXiv preprint arXiv:1707.02937 (2017)

[7] Fang, Q., Boas, D.A.: Tetrahedral mesh generation from volumetric binary and grayscale images. In: 2009 IEEE International Symposium on Biomedical Imaging:

from Nano to Macro, pp. 1142–1145 (2009). Ieee

[8] Fey, M., Lenssen, J.E.: Fast graph representation learning with PyTorch Geometric. In: ICLR Workshop on Representation Learning on Graphs and Manifolds (2019)

[9] Glorot, X., Bengio, Y.: Understanding the difficulty of training deep feedforward neural networks. In: Proceedings of the Thirteenth International Conference on Artificial Intelligence and Statistics, pp. 249–256 (2010). JMLR Workshop and Conference Proceedings
