# Supplementary material for: Deep-Motion-Net: GNN-based volumetric liver shape reconstruction from single-view 2D projections
Source: Int J Comput Assist Radiol Surg. 2026 May 13;21(6):1253–62. doi: 10.1007/s11548-026-03685-1 (PMC13451316; doi:10.1007/s11548-026-03685-1)
Supplement: Supplementary file 2 [file 11548_2026_3685_MOESM2_ESM.docx]

**Online Resource 2 (Supplementary): Deep-Motion-Net: GNN-based volumetric organ shape reconstruction from single-view 2D projections**

| Article Title | Deep-Motion-Net: GNN-based volumetric organ shape reconstruction from single-view 2D projections |
| --- | --- |
| Conference Name | International Conference on Information Processing in Computer-Assisted Interventions |
| Journal Name | International Journal of Computer Assisted Radiology and Surgery |
| Affiliation | University of Leeds |
| Email Address | isurusuranga.wijesinghe@gmail.com |

**Real kV Evaluation – animation – Second scan series - GNN (Patient 1)**

Animation for full-scan-series with predicted contours overlaid on corresponding real kV images

**Real kV Evaluation – animation – Second scan series - GNN (Patient 2)**

Animation for full-scan-series with predicted contours overlaid on corresponding real kV images
